# Supplementary figures and images for: Liver-specific glucagon dysfunction promotes PP-cell hyperplasia and formation of glucagon and PP double-positive cells
Source: PLoS One. 2025 Sep 23;20(9):e0329094. doi: 10.1371/journal.pone.0329094 (PMC12456763; doi:10.1371/journal.pone.0329094)

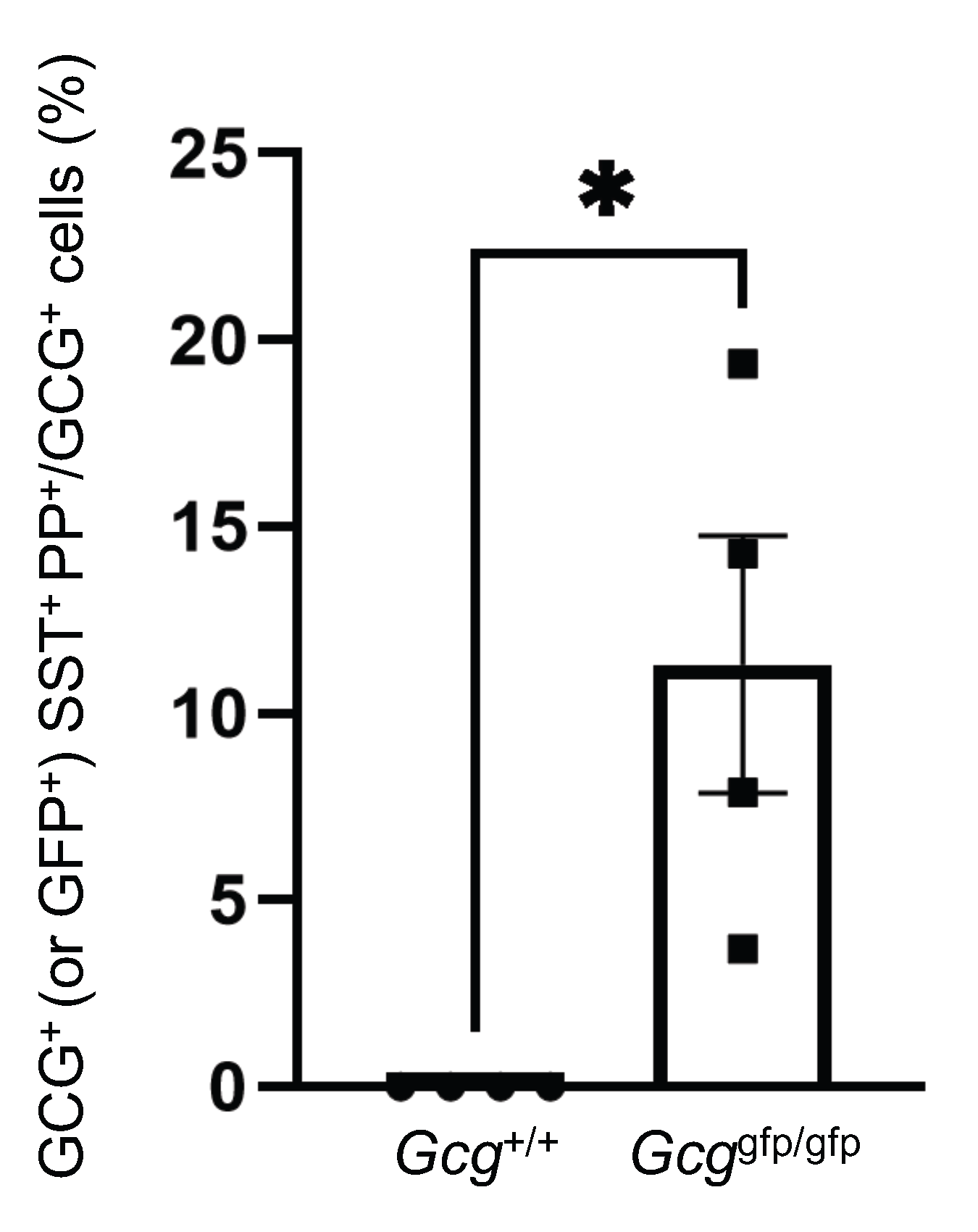

Supplement: S1 Fig — Data are shown as the mean ± SEM, and analyzed by the two-tailed unpaired Student t-test. *p < 0.05; n = 4 each. (TIF) [file pone.0329094.s001.tif]

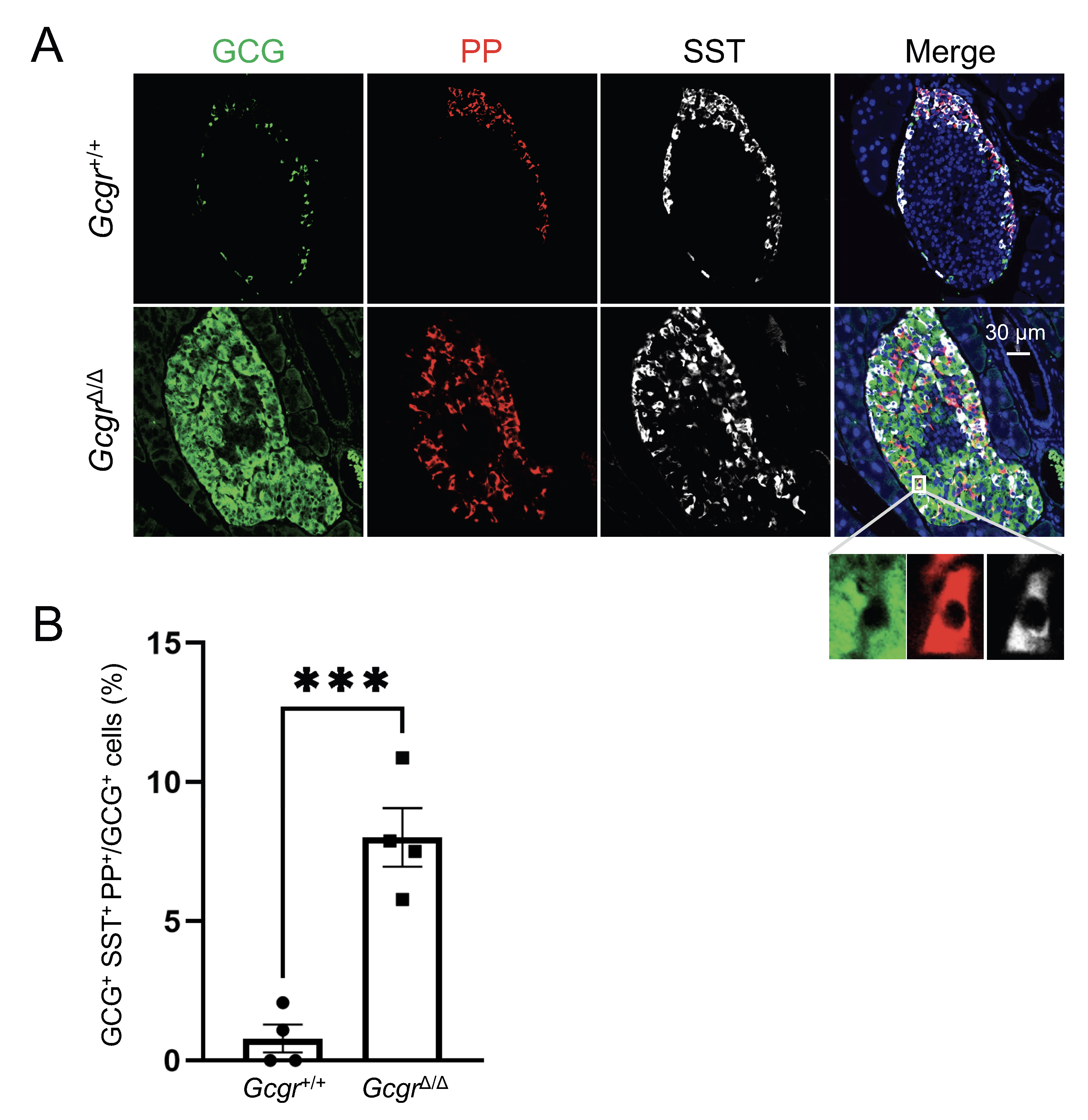

Supplement: S2 Fig — (A) Representative immunofluorescence staining of GCG (green), SST (red), PP (white), and nuclei (DAPI; blue) in pancreatic sections from 10-week-old Gcgr+/+ mice and GcgrΔ/Δ mice. Magnified images of the boxed region are shown at the bottom. Scale bar represents 30 μm. (B) Quantification of the proportion of GCG ⁺ SST ⁺ PP ⁺ cells among the GCG⁺ cells in 10-week-old Gcgr+/+ mice and GcgrΔ/Δ mice (n = 4 each). Data are shown as the mean ± SEM, and analyzed by the two-tailed unpaired Student t-test. ***p < 0.001. (TIF) [file pone.0329094.s002.tif]

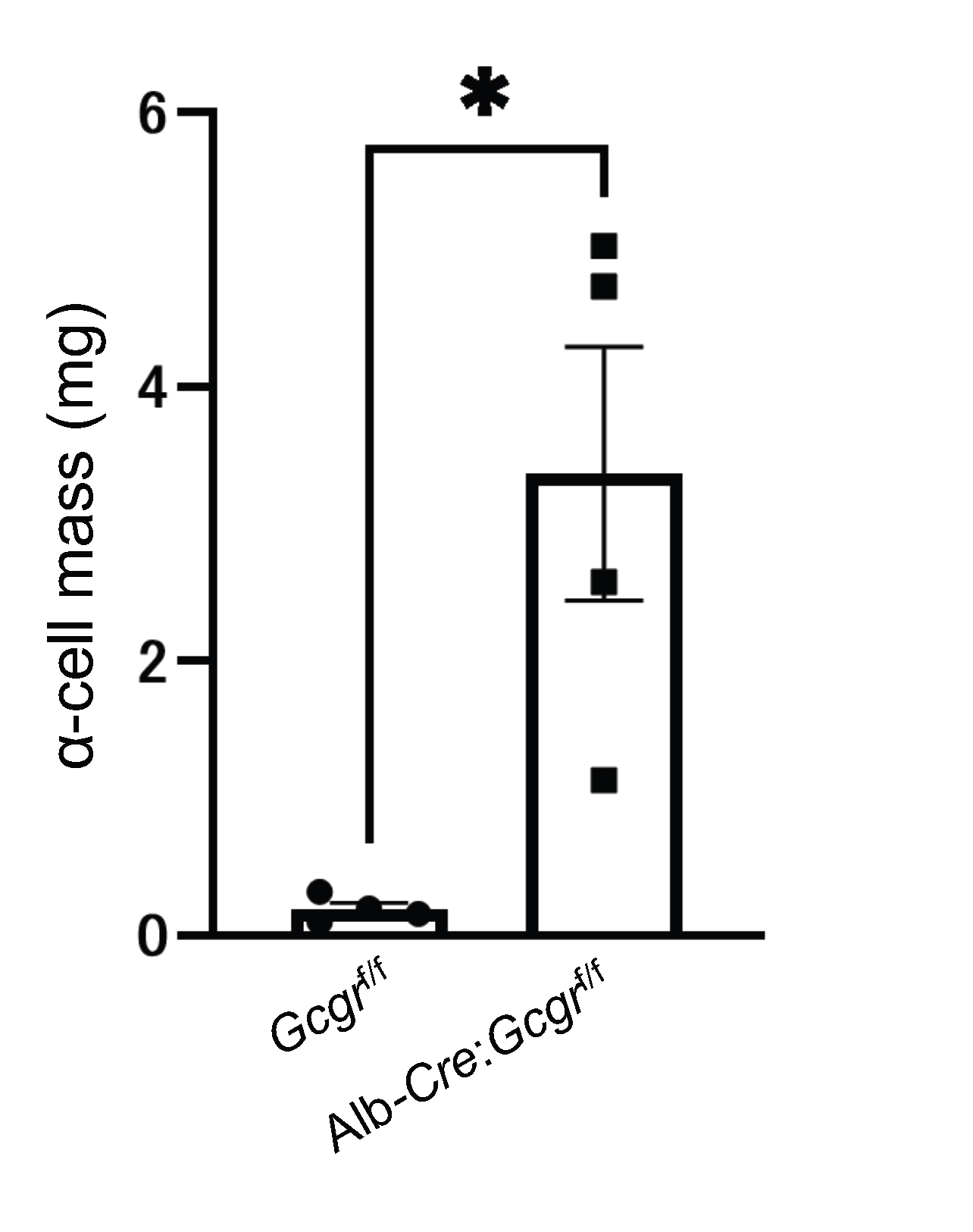

Supplement: S3 Fig — Data are shown as the mean ± SEM, and analyzed by the two-tailed unpaired Student t-test. *p < 0.05; n = 4 each (TIF) [file pone.0329094.s003.tif]

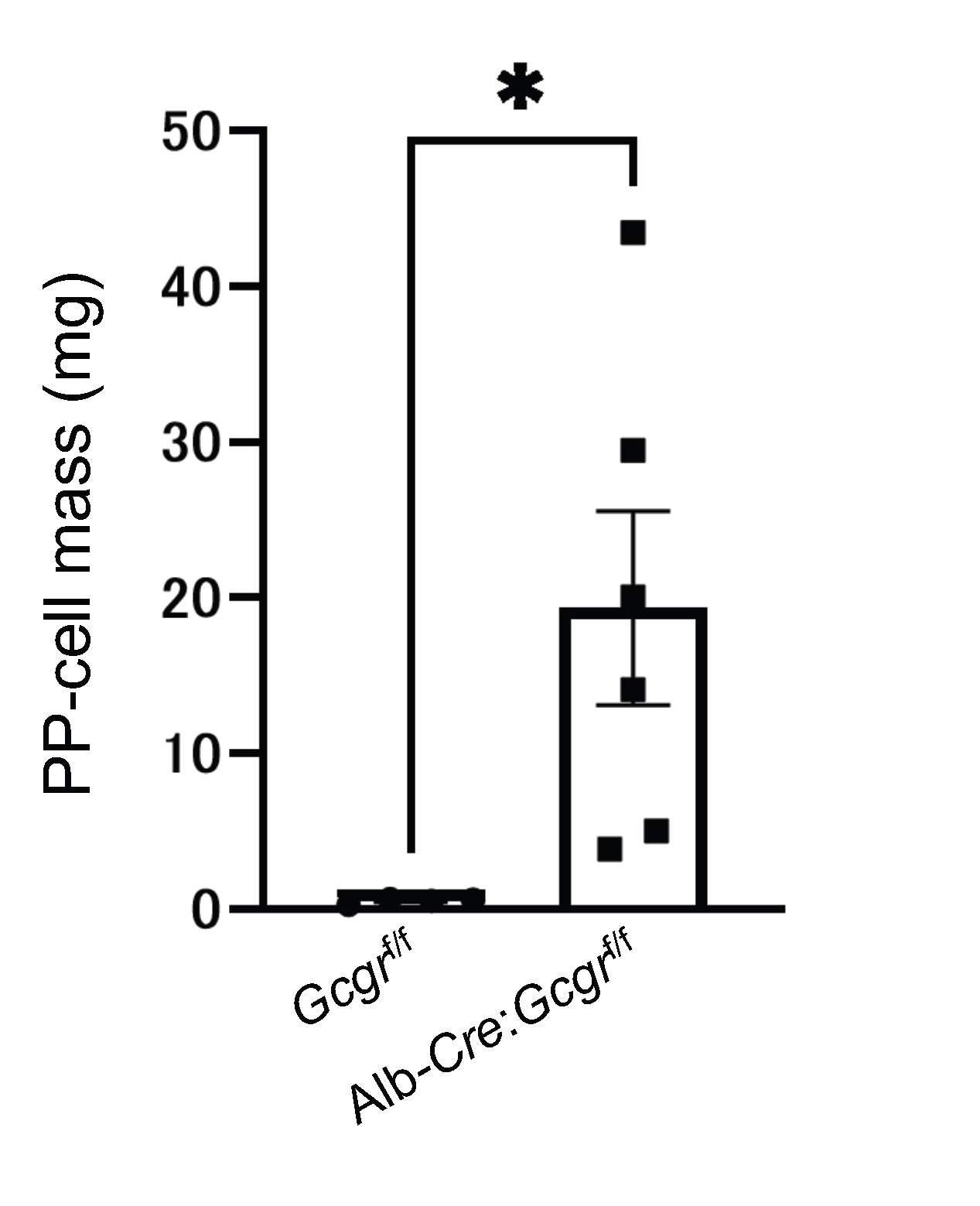

Supplement: S4 Fig — Data are shown as the mean ± SEM, and analyzed by the two-tailed unpaired Student t-test. *p < 0.05; n = 6 each. (TIF) [file pone.0329094.s004.tif]

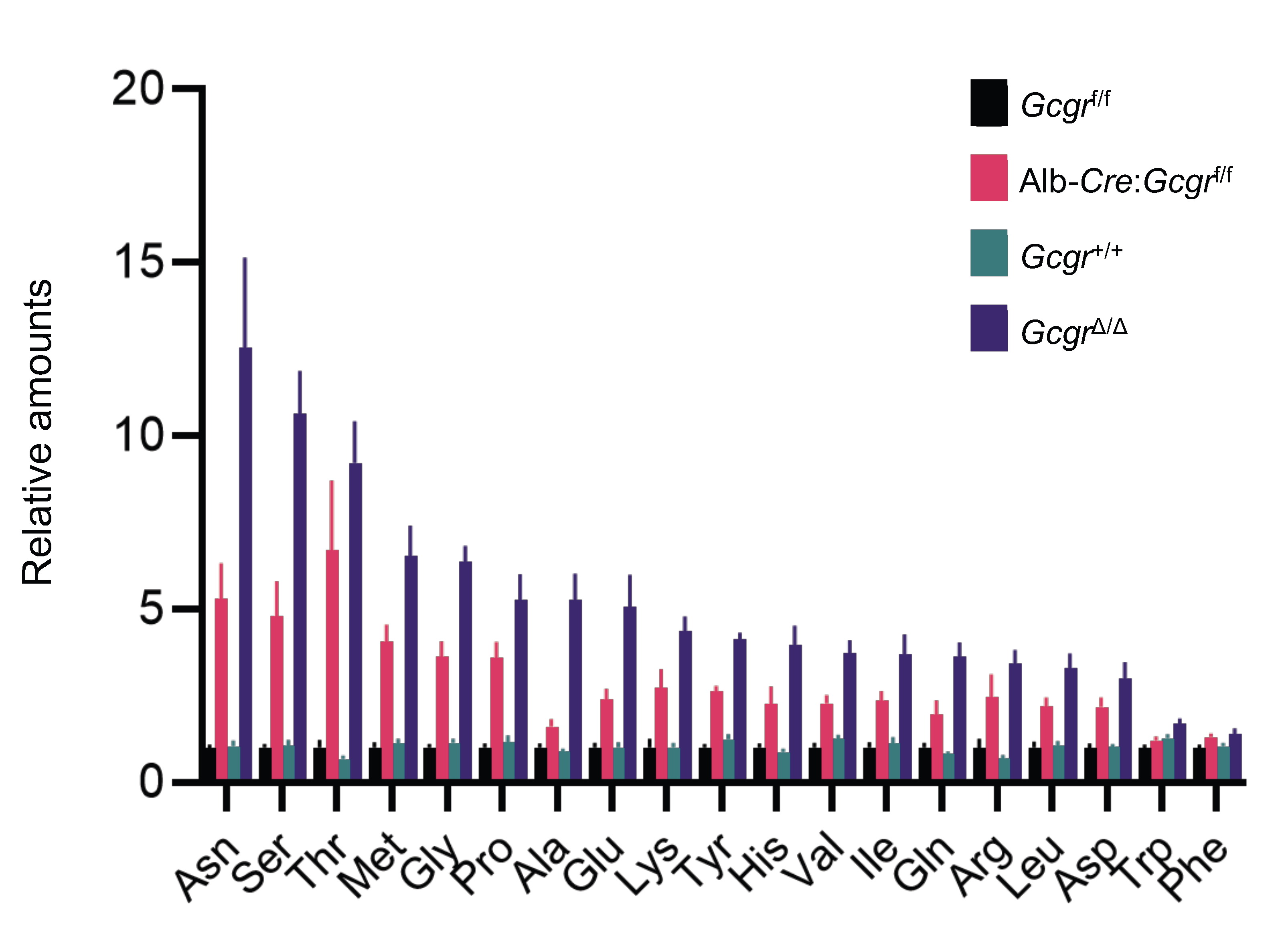

Supplement: S5 Fig — The relative amounts shown on the Y-axis represent normalized values, where the mean Gcgrf/f level for each amino acid is set to 1. Therefore, it is not appropriate to directly compare these values between different amino acids. Data are shown as the mean ± SEM (n = 4–7 mice each). (TIF) [file pone.0329094.s005.tif]

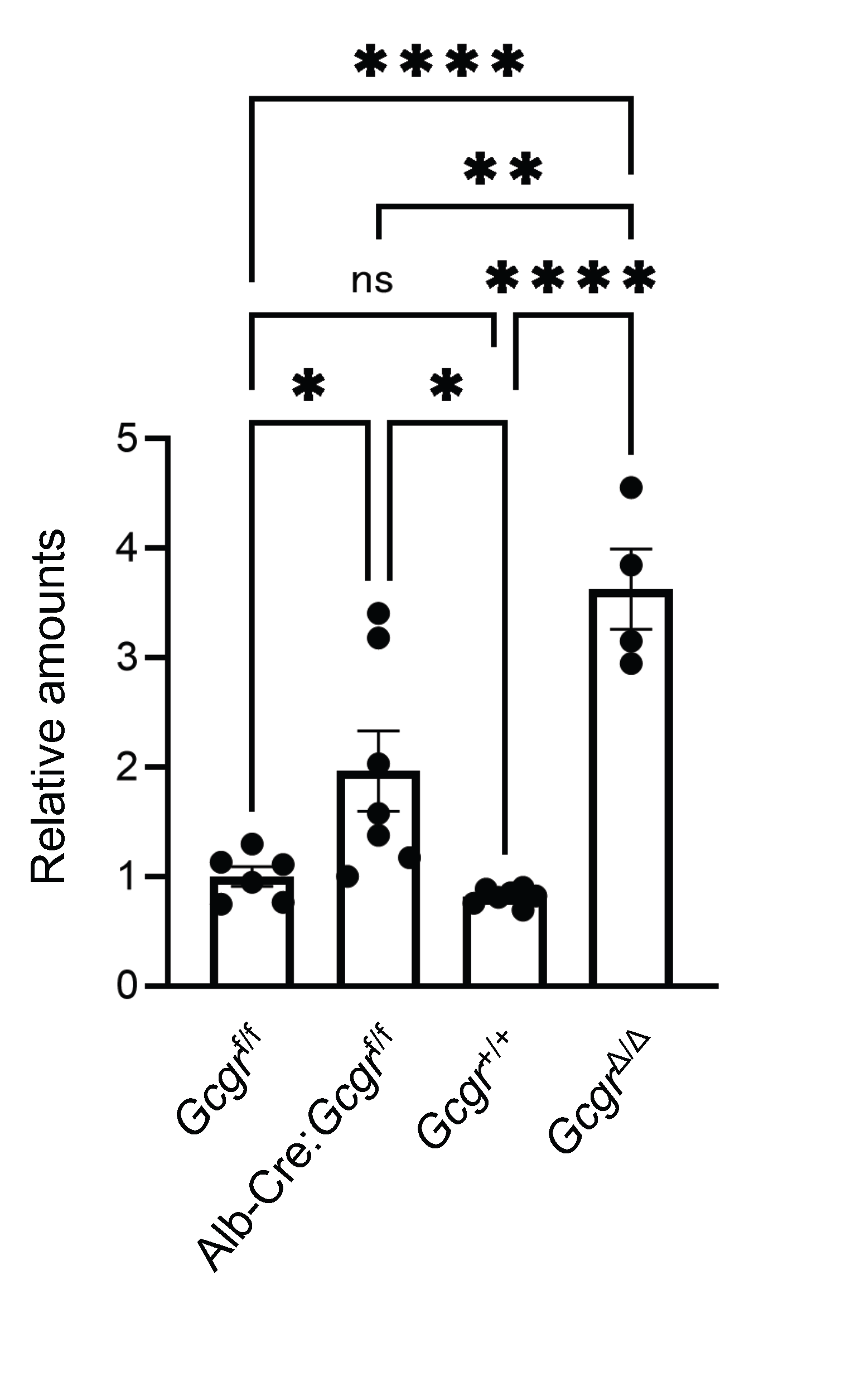

Supplement: S6 Fig — The relative amounts shown on the Y-axis represent normalized values, where the mean Gcgrf/f level is set to 1. Data are shown as the mean ± SEM, and analyzed by one-way ANOVA followed by the Tukey test. *p < 0.05, **p < 0.01, ****p < 0.0001; ns, not significant. (TIF) [file pone.0329094.s006.tif]

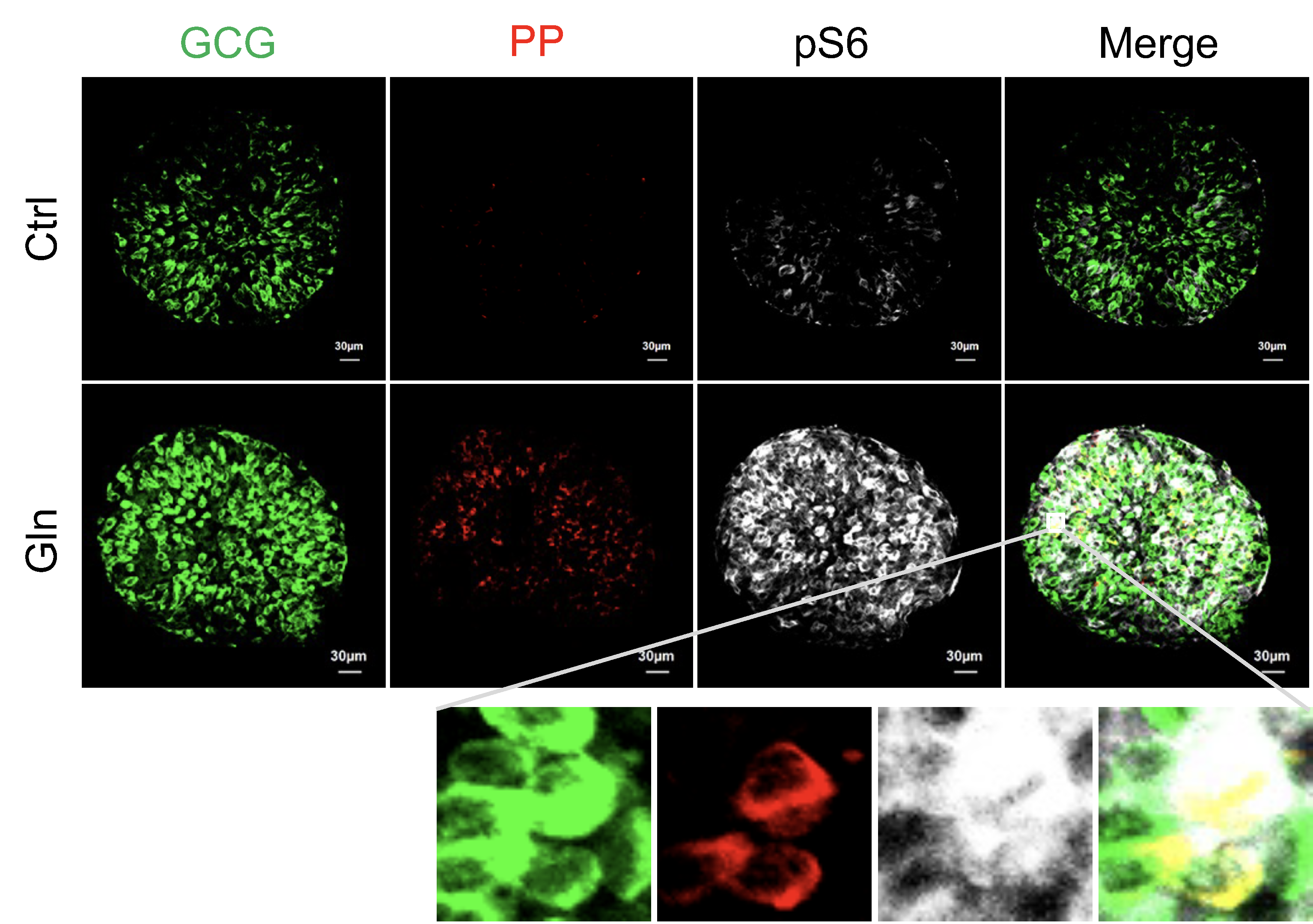

Supplement: S7 Fig — Representative immunofluorescence images of pancreatic islets isolated from 10-week-old wild-type mice stained for GCG (green), PP (red), and pS6 (white). Upper panels show untreated controls (Ctrl), and lower panels show islets incubated with high concentrations of glutamine (Gln). Scale bar: 30 μm. Enlarged views of the boxed regions are shown at the bottom. (TIF) [file pone.0329094.s007.tif]

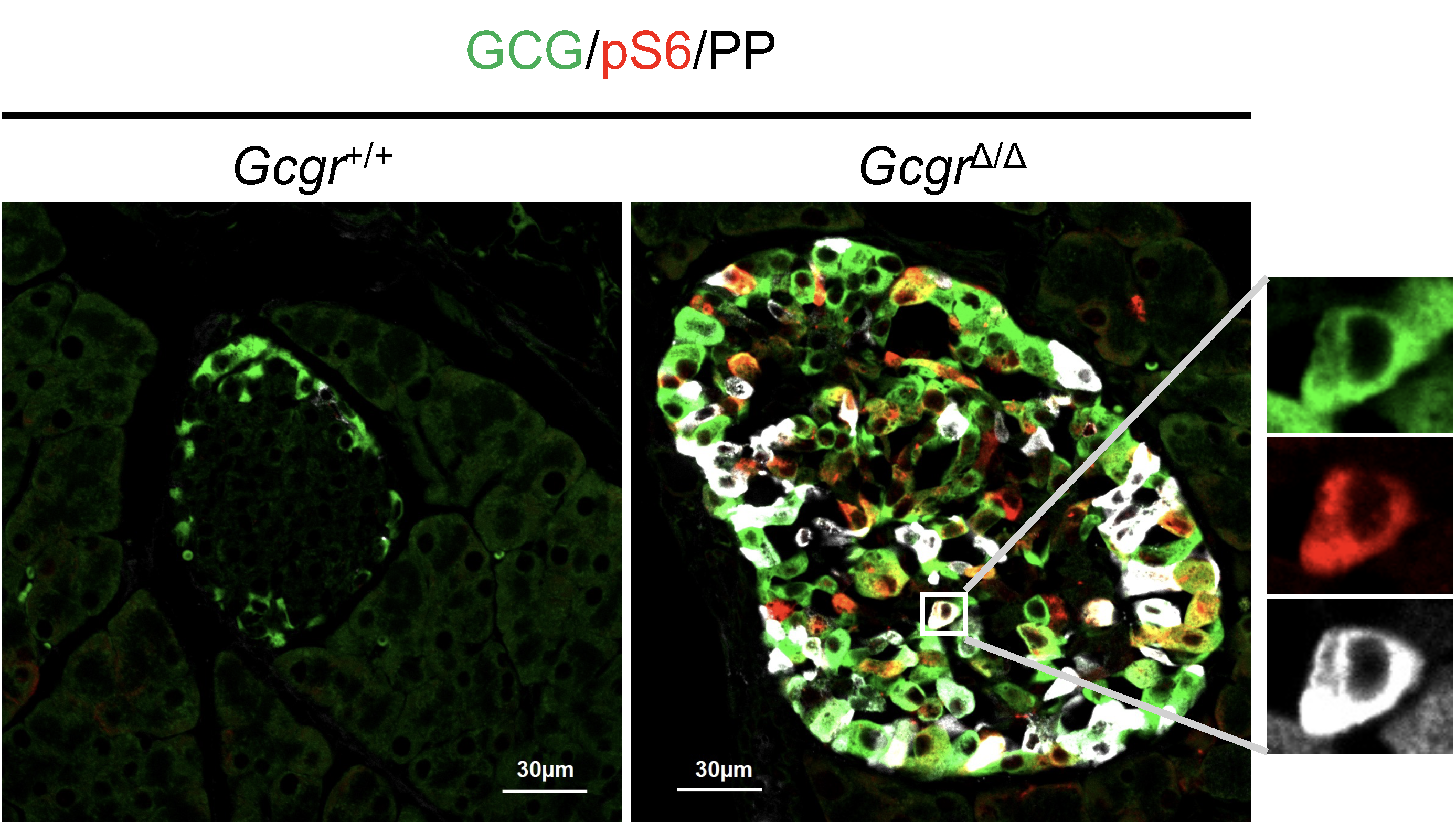

Supplement: S8 Fig — Representative immunofluorescence staining of GCG (green), pS6 (red), and PP (white) from 40-week-old Gcgr+/+ mice and GcgrΔ/Δ mice. Scale bar represents 30 μm. Enlarged images of the boxed region are shown on the very right. (TIF) [file pone.0329094.s008.tif]

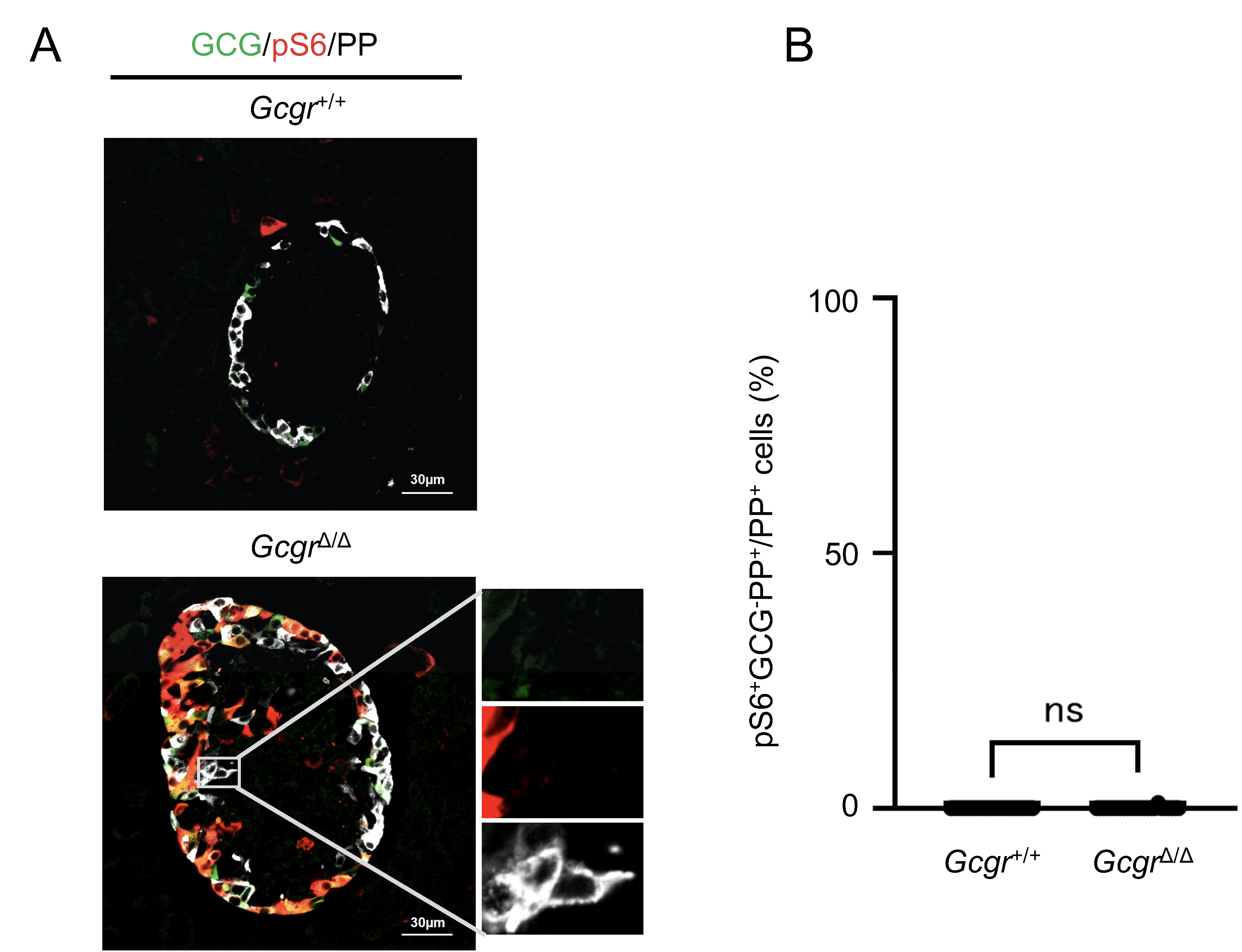

Supplement: S9 Fig — (A) Representative immunofluorescence staining of GCG (green), pS6 (red), and PP (white) from 4-week-old Gcgr+/+ mice and GcgrΔ/Δ mice. Scale bar represents 30 μm. Enlarged images of the boxed region are shown on the very right. (B) Ratio of pS6+ GCG− PP+ cells per total PP+ cells in 4 week-old Gcgr+/+ mice and GcgrΔ/Δ mice (n = 25 islets). Data are shown as the mean ± SEM, and analyzed by the two-tailed unpaired Student t-test. ns, not significant. (TIF) [file pone.0329094.s009.tif]

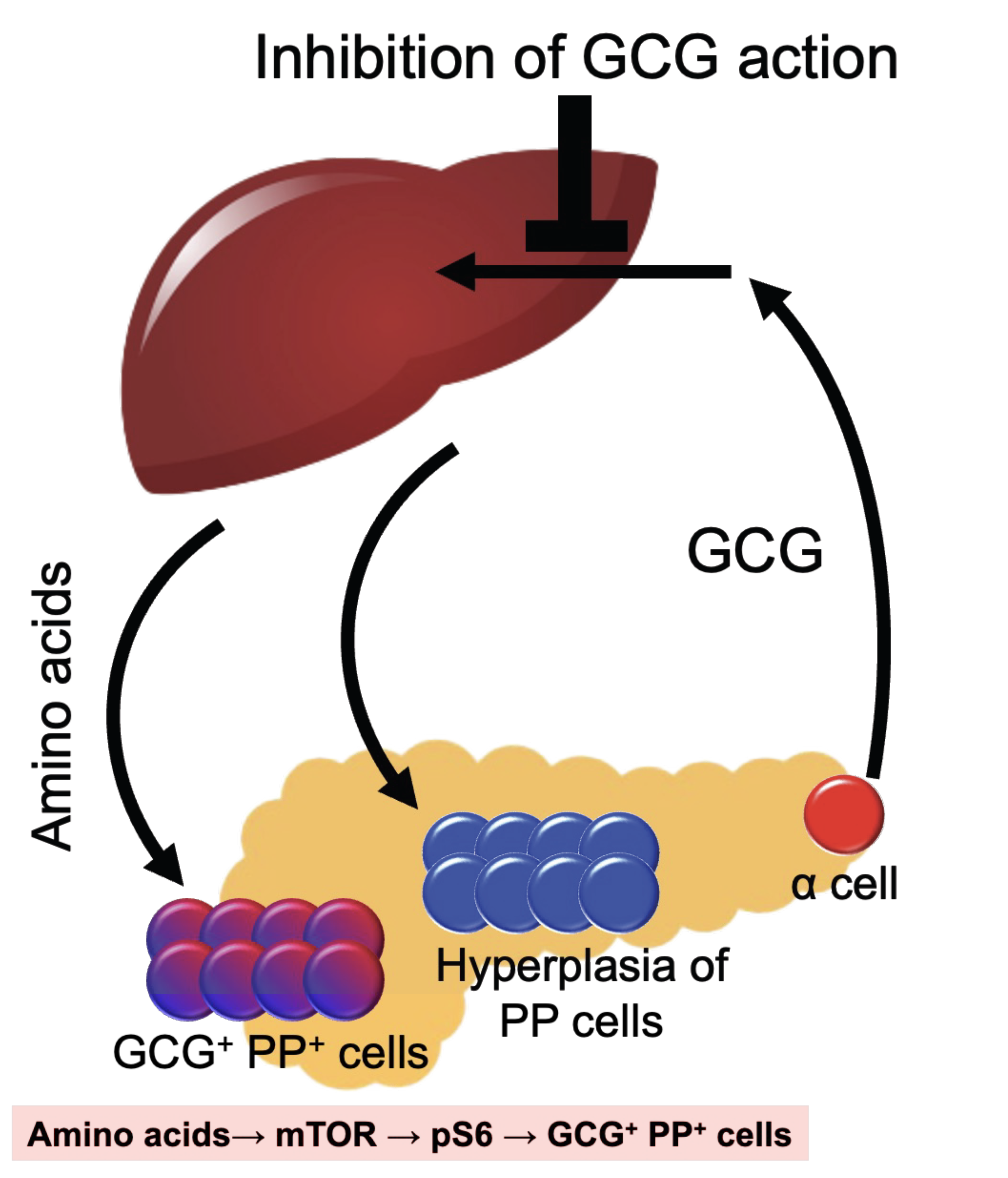

Supplement: S10 Fig — Impaired glucagon action in the liver leads to increased circulating amino acid levels, which in turn activate the mTOR signaling pathway in pancreatic α cells, promoting α-cell hyperplasia and an increase in GCG ⁺ PP⁺ cells. Simultaneously, this condition also induces the hyperplasia of pancreatic PP cells. (TIF) [file pone.0329094.s010.tif]
