# Supplementary material for: Liver-specific glucagon dysfunction promotes PP-cell hyperplasia and formation of glucagon and PP double-positive cells
Source: PLoS One. 2025 Sep 23;20(9):e0329094. doi: 10.1371/journal.pone.0329094 (PMC12456763; doi:10.1371/journal.pone.0329094)
Supplement: Attachment — Submitted filename: Response to Reviewers Fujitani 4.docx [file pone.0329094.s011.docx]

Please find our point-by-point responses (in blue) to the comments of the reviewers below.

**Point-by-point responses to Reviewer #1**

**Comments:**

**Reviewer 1**

Comment 1:

In the Methods section, under "Immunohistochemistry," the fixation method through perfusion is described. However, there is an inconsistency between this description and the handling of tissues in the "Quantification of α- and PP-cell mass" section. Please revise the manuscript to ensure consistency between these two sections.

Response 1:

Thank you very much for pointing out this inconsistency. As you correctly noted, the same perfusion-fixation method was used for both the immunohistochemistry analysis and the quantification of α-cell and PP-cell mass. However, the description of the latter lacked sufficient detail. We have now revised the “Quantification of α-cell and PP-cell mass” section to clearly indicate that cardiac perfusion was performed with normal saline followed by 4% paraformaldehyde, in accordance with the procedure outlined in the “Immunohistochemistry” section (please refer to page 13, lines 176–179 of the revised manuscript). We hope this clarification resolves the inconsistency. We have listed the revised sections of the manuscript below for your kind reference.

**Quantification of α-cell and PP-cell mass**

The quantification of α-cell mass and PP-cell mass was performed as previously described [24, 33]. After anesthesia with isoflurane, mice were perfused through the heart with normal saline followed by 4% PFA, as described in the “Immunohistochemistry” section. Pancreata were then harvested, and nonpancreatic tissues were removed. The pancreata were post-fixed in 4% PFA at 4 °C overnight. To obtain longitudinal sections from the tail to the head of the pancreas, the pancreas was embedded in paraffin, and paraffin blocks were prepared. From the entire pancreas, 6 sections per mouse were collected at 200-µm intervals, and immunostained with an anti-GCG or anti-PP antibody.

Comment 2:

In the "Islet culture" section, there is insufficient information about how islets were collected. A brief additional explanation would be helpful. Also, at the end of this section, the embedding with OCT compound is mentioned, but there is no further description about immunostaining. Please either add this explanation to this section or include it in the "Immunohistochemistry" section.

Response 2:

Thank you for your valuable feedback. As you pointed out, the methods for immunostaining and the details of the antibodies used in this study were not included in the original manuscript. We have now incorporated this information into the main text (please refer to pages 14-15, lines 201–202 of the revised manuscript). We hope this addition addresses your concerns and meets your expectations. We have listed the revised sections of the manuscript below for your kind reference.

Finally, frozen tissue blocks were prepared using Optimal Cutting Temperature compound, followed by cryosectioning [32]. Details of the immunostaining procedure and antibodies used are provided in the “Immunohistochemistry” section above. The sections were subsequently visualized using an FV1000 confocal laser scanning microscope (Olympus).

Comment 3:

In the "Metabolomic analysis" methods, there is no explanation about which tissues' "frozen samples" were used, and the Results section does not mention the results obtained using this method. This should be clarified and included.

Response 3:

**Thank you for your comment. The term “frozen sample” refers to the plasma sample described just before the Methods section. We apologize for the lack of clarity in our initial description. This has been corrected in the revised manuscript (pages 15-16, lines 217–218).** **In response to your concern that data using this method do not appear in the Results section, we would like to clarify that the relevant data are presented in S5 Fig and S6 Fig, as described on page 26, line 374 of the revised manuscript. We hope this clarifies this issue.** We have listed the revised sections of the manuscript below for your kind reference.

**Metabolomics analysis**

Widely targeted metabolomics analysis was performed as previously described [35, 36]. Briefly, frozen plasma samples (the sample preparation method is described above in the “Mouse plasma collection” section) in 1.5-mL plastic tubes were homogenized in 300 µL of cold methanol using φ3-mm zirconia beads and a freeze crusher (TAITEC Corp., Saitama, Japan) at 41.6 Hz for 2 min. The homogenates were mixed with 200 µL of methanol, 200 µL of H_2_O, and 200 µL of CHCl_3_, and vortexed for 20 min at room temperature. The samples were then centrifuged at 15,000 rpm (20,000 *g*) for 15 min at 4 ℃.

Comment 4:

In Figure 2B, the ratio of Gcg/PP double-positive cells is quantified. It would be helpful to perform the same quantification in Figure 1B for consistency. Additionally, in Figure 1B, SST staining is shown; please include SST staining in Figure 2B as well and provide the ratios of single, double, and triple-positive cells for each marker.

Response 4:

Thank you for your valuable comments. We have added the quantitative data for Figure 1B to S1 Fig. In addition, we performed triple immunostaining using GCG, PP, and SST antibodies on samples from the same time point as in Figure 2B, and quantified the proportion of GCG⁺ PP⁺ SST⁺ cells (S2 Fig) (please refer to page 18, line 257 and page 20, lines 282–285 of the revised manuscript). The newly created figures and the revised sections of the manuscript are attached below. We would appreciate it if you could kindly review them.

**S2 Fig. Triple immunostaining using anti-GCG, anti-PP, and anti-SST antibodies in pancreatic islets of 10-week-old *Gcgr^+/+^* mice and *Gcgr*^Δ/Δ^ mice**

**S1 Fig. Proportion of GCG⁺ SST^+^ PP⁺ cells among GCG⁺ cells in 40-week-old *Gcg^+/+^* mice and *Gcg^gfp/gfp^* mice**

Quantitative analysis demonstrated a significant increase in both PP-cell mass and the ratio of GCG^+^ PP^+^ cells relative to GCG^+^ cells in *Gcgr*^Δ/Δ^ mice compared with *Gcgr*^+/+^ mice (Figs 2C and D). Furthermore, PP^+^ GCG^+^ SST^+^ cells were also detected in *Gcgr*^Δ/Δ^ mice at this stage, consistent with the findings shown in Fig 1B (S2 Fig). Collectively, these findings indicate that GCG signaling deficiency induces PP-cell hyperplasia and induces the formation of multi-hormonal cells, including GCG^+^ PP^+^ cells.

Comment 5:

In the lines after 305, the term "self-proliferation" is used multiple times, but since it has not been evaluated whether the proliferation is truly "self" or not, it would be more appropriate to simply use the term "proliferation."

Response 5:

Thank you very much for your valuable suggestion. In accordance with the suggestion, we have replaced all six instances of the term “self-proliferation” with “proliferation” in the revised manuscript (please refer to page 22, lines 311, 313 and 321; page 30, lines 435, 440-442). In addition, the term “self-replication” has also been revised to “replication” to improve clarity and for consistency (please refer to page 2, line 32; page 21, line 296; and page 22, line 317).

Comment 6:

In Figures 3D and 3F, the ratios are used for quantification. However, as with Figure 3B, showing mass or the ratio relative to the total islet cells would help enhance the reader's understanding.

Response 6:

**Thank you very much for your valuable comments. We would first like to address the point regarding Fig 3F. This experiment was designed to evaluate whether PP cells that do not express GCG are proliferating in *Gcgr*^Δ/Δ^ mice at 4 weeks of age, a stage at which GCG⁺ PP⁺ cells have not yet become prominent. Therefore, rather than assessing overall PP-cell mass or the ratio of Ki67⁺ cells relative to the total islet cell population, we believe that the most appropriate and direct approach is to calculate the proportion of Ki67⁺ cells specifically among PP cells that do not co-express glucagon. This analysis enables a more accurate assessment of proliferation within the native PP-cell population. Regarding Fig 3D, as shown in Fig 3C, a comparison between 4-week-old and 10-week-old mice demonstrated that whereas the number of GCG⁺ cells remains high at both time points, the number of PP cells increases substantially by 10 weeks. Notably, GCG⁺-positive cells already account for approximately 70% to 80% of the islet cell population at 4 weeks, and this proportion remains relatively stable up to 10 weeks. Based on this observation, we hypothesized that the apparent increase in PP cells at 10 weeks may be at least partially owing to the emergence of GCG^+^ cells that newly begin to co-express PP. To test this possibility, we chose to evaluate the proportion of GCG⁺ PP⁺ cells within the total GCG^+^-positive population, as shown in Fig 3D. We believe this method provides a more accurate representation of phenotypic changes within the GCG^+^ cell population, and appropriately reflects the underlying biological processes.**

Comment 7:

In Figure 5F, including the Ctrl+Rap group would make the comparison with the control group clearer.

Response 7:

Thank you for your comment. Previous studies have shown that whereas rapamycin suppressesα-cell proliferation in GCG receptor-deficient mice showing hyperplasia, it does not affect α-cell numbers in wild-type mice [1]. Therefore, in the present study, we did not administer rapamycin to islets from wild-type mice.

Reference

**1.**	Solloway MJ, Madjidi A, Gu C, Eastham-Anderson J, Clarke HJ, Kljavin N, et al. Glucagon Couples Hepatic Amino Acid Catabolism to mTOR-Dependent Regulation of alpha-Cell Mass. Cell Rep. 2015; 12(3): 495-510. <https://doi.org/10.1016/j.celrep.2015.06.034>. PMID: 26166562.

**Point-by-point responses to Reviewer #2**

**Comments:**

**Reviewer 2**

Comment 1:

The authors identify GCG+ PP+ and PP+ INS+ cells but do not assess whether these cells are functional. Do they secrete both hormones?
Authors can try hormone secretion assays like ELISA for Glucagon or PP after sorting the cells.

Response 1:

Thank you very much for your insightful and thought-provoking comment. We are also deeply interested in elucidating the cellular characteristics of GCG⁺ PP⁺ and PP⁺ INS⁺ cells. However, to date, no specific surface markers have been identified that can reliably distinguish these two double-positive populations. As a result, isolating these cells through sorting remains unfeasible at present. In the future, we aim to identify surface markers unique to each population, which will enable us to isolate them and thoroughly characterize their functional properties. We have acknowledged this as a limitation in the revised manuscript (please see page 34, lines 498–504). For your convenience, the revised sections of the manuscript are listed below for your kind reference.

Another limitation is our inability to characterize the cellular properties of GCG⁺ PP⁺ and PP⁺INS⁺ cells. To date, no specific surface markers have been identified that can reliably distinguish these double-positive cell populations. As a result, isolating them by cell sorting remains unfeasible. In future studies, identifying surface markers unique to each population will be essential for isolating these cells and comprehensively analyzing their functional characteristics. Such advances will be crucial for understanding the physiological relevance of these distinct endocrine cell populations.

Comment 2:

The origin of double-positive cells remains unclear. Are α cells adopting PP identity, or are they a distinct lineage?

Response 2:

Thank you very much for your insightful question. We have also been deeply interested in this issue. Although the Cre-loxP system we employed in this study is widely used, it has limitations in labeling specificity, and off-target labeling can occur, making it difficult to accurately determine the origin of the GCG^+^ PP^+^ cells. In contrast, a recently developed **dual-recombinase-mediated intersectional genetic strategy** enables the selective labeling of cells that coexpress two specific genes [2]. We believe that this approach holds promise for elucidating the origin of GCG^+^ PP^+^ cells. However, implementing this strategy would require the generation of new genetically engineered mouse lines, which is not feasible within the timeframe of this study’s revision period, and we hence ask for your kind understanding on this matter. Therefore, the inability to determine the origin of GCG^+^ PP^+^ cells remains one of the limitations of this work. We have mentioned this as a limitation in the revised manuscript. (please refer to pages 33-34, lines 494–498 of the revised manuscript). We have listed the revised sections of the manuscript below for your kind reference.

One of the limitations of this study is the inability to determine the origin of GCG⁺PP⁺ cells. Although the Cre-loxP system employed here is widely used for lineage tracing, it has inherent limitations in labeling specificity. Off-target recombination can occur, which complicates the precise identification of cell origin. Consequently, we were unable to definitively trace the developmental lineage of GCG⁺PP⁺ cells using this approach.

Reference

**2.**	Huang X, Zhao H, Chen H, Liu Z, Liu K, Lv Z, et al. Ductal or Ngn3(+) cells do not contribute to adult pancreatic islet beta-cell neogenesis in homeostasis. EMBO J. 2025. <https://doi.org/10.1038/s44318-025-00434-z>. PMID: 40205162.

Comment 3:

While mTOR activation is implicated, the downstream effectors remain undefined. Authors should check at least mRNA expression of mTOR pathway targeted genes.

Response 3:

Thank you very much for your important comment. In response to your suggestion, we conducted further analysis and confirmed that, consistent with previous reports on α cells in GCG signaling-deficient mice [1], the GCG⁺ PP⁺ cells induced by glutamine also show the phosphorylation of S6, a downstream effector of the mTOR pathway. These findings suggest that glutamine activates the mTOR signaling pathway in GCG⁺ PP⁺ cells, specifically leading to the activation of its downstream effector, S6. This indicates that protein synthesis and cell proliferation may be increased in these cells. The corresponding data have been added as Supplementary Figure 7, and an explanation has also been added to the text (please refer to page 26, lines 379–384 of the revised manuscript). The newly created figures and the revised sections of the manuscript are attached below. We would appreciate it if you could kindly review them.

**S7 Fig. Triple immunostaining of pancreatic islets from 10-week-old wild-type mice using anti-GCG, anti-PP, and anti-pS6 antibodies**

Moreover, the ratio of GCG^+^ PP^+^ cells among GCG^+^ cells also increased (Fig 5D). To determine whether the increase in GCG⁺ PP⁺ cells was mediated by the mTOR pathway, pancreatic islets were treated with the mTOR kinase inhibitor rapamycin. The glutamine-induced increase in GCG⁺ PP⁺ cells was significantly suppressed by rapamycin treatment (Figs 5E and F). We then investigated whether pS6, a downstream target of mTOR signaling, could be detected in GCG⁺ PP⁺ cells under glutamine-rich conditions. As shown in S7 Fig, pS6 signals were clearly observed in these cells.

Reference

**1.**	Solloway MJ, Madjidi A, Gu C, Eastham-Anderson J, Clarke HJ, Kljavin N, et al. Glucagon Couples Hepatic Amino Acid Catabolism to mTOR-Dependent Regulation of alpha-Cell Mass. Cell Rep. 2015; 12(3): 495-510. <https://doi.org/10.1016/j.celrep.2015.06.034>. PMID: 26166562.

Comment 4:

There is no information about the sex of the mice. Did the study design with both mice? If both types of mice are used for the study, then author should observe whether the phenotypes are sex dependent or not?

Response 4:

Thank you for your comment. All experiments in this study were performed using male mice. This information has been added to the “Mice” section of Materials and Methods (please refer to page 9, line 123 of the revised manuscript). We have listed the revised sections of the manuscript below for your kind reference.

**Mice**

B6D2F1 mice were purchased from CLEA Japan (Kawasaki, Japan), ICR mice were obtained from Charles River Japan (Yokohama, Japan), and C57BL/6JmsSlc mice were obtained from Japan SLC, Inc. (Shizuoka, Japan). Male mice were used for all the experiments.

Comment 5:

Provide a graphical model summarizing the proposed mechanism.

Response 5:

Thank you for your suggestion. We have added a graphical summary of this study as Supplementary Figure 10 (please refer to page 34, line 507 of the revised manuscript). The newly created figure of the manuscript is attached below. We would appreciate it if you could kindly review them.

**S10 Fig. Graphical abstract of the study**

Comment 6:

Although the current title is technically accurate, it is somewhat complex and may be difficult for readers to grasp easily. I recommend simplifying the title to enhance clarity and accessibility, particularly for a broad, interdisciplinary readership. Consider rephrasing to more directly convey the central findings, such as: “Liver-specific glucagon dysfunction promotes PP-cell hyperplasia and formation of glucagon+ PP+ endocrine cells.

Response 6:

Thank you very much for your excellent suggestion. We had not thought of such a clear and concise title ourselves, and we greatly appreciate your insight. We have revised the title accordingly, and are sincerely grateful for your valuable input.
